# Comparison of Perioperative, Functional, and Oncological Outcomes of Transperitoneal and Extraperitoneal Laparoscopic Radical Prostatectomy

**DOI:** 10.1155/2023/3263286

**Published:** 2023-02-07

**Authors:** Tanan Bejrananda, Watid Karnjanawanichkul, Monthira Tanthanuch

**Affiliations:** Division of Urology, Department of Surgery, Faculty of Medicine, Prince of Songkla University, Hatyai, Songkhla, Thailand

## Abstract

**Purpose:**

This study aimed to compare the oncological, functional, and perioperative outcomes of localized and locally advanced prostate cancer treated with intraperitoneal or extraperitoneal laparoscopic radical prostatectomy (LRP).

**Methods:**

From April, 2008, through December, 2020, 266 patients underwent laparoscopic radical prostatectomy, 168 cases with an extraperitoneal approach (E-LRP) and 98 cases using a transperitoneal approach (T-LRP). The clinical, perioperative, functional, and oncological outcomes were collected and compared between these groups. At the 3-, 12- and 24-monthfollow-ups, the functional outcomes tested were urinary function (urinary domain of EPIC) and sexual function (sexual domain of EPIC). The oncological outcomes of biochemical recurrence, biochemical recurrence-free survival, and positive surgical margin status were evaluated. Univariable and multivariable Cox regression analyses were used to identify factors predictive for biochemical recurrence. All statistical analyses used the R program.

**Results:**

The patient characteristics were similar between the E-LRP and T-LRP groups except for higher prostatic-specific antigen (PSA) in the T-LRP group. The T-LRP had lower overall operative time (222.5 min vs. 290 min, *p* 0.001), decreased blood loss (400 ml vs. 800 ml, *p* < 0.001), and shorter hospital stays (4 days vs. 7 days, *p* < 0.001) compared to the E-LRP. Early sexual intercourse with penetration at 3 months was higher in the T-LRP group (36.7% vs. 15.5%, *p* 0.001). Urinary continence (no pads) was not different between the T-LRP and E-LRP groups at 3 and 24 months after surgery but higher in the E-LRP group at 12 months (1% vs. 3%; *p*=0.419, 85.1 vs. 83.7%; *p*=0.889, 47.4% vs. 34.6%; *p*=0.028, respectively). The EPIC questionnaire was used to assess functional outcomes at 3, 12, and 24 months after surgery and found that urinary function was significantly higher in the T-LRP group at 3 and 12 months (*p* < 0.001) but did not show a difference at 24 months (*p*=0.734), and sexual function scores were higher in the T-LRP group at 12 and 24 months (*p*=0.001). The positive surgical margin rate was higher in the E-LRP (38.7% vs. 21.4%; *p*=0.006). The BCR rate was not different between the groups (36.3% in the E-LRP group and 27.6% in the E-LRP group; *p*=0.184).

**Conclusion:**

Transperitoneal laparoscopic radical prostatectomy (T-LRP) was found to be superior to extraperitoneal radical prostatectomy (E-LRP) in perioperative outcomes such as decreased operative time, decreased blood loss, shorter hospital stay, lower positive surgical margin, and improved early sexual intercourse and sexual function. The urinary functional outcome was better in the T-LRP group at 3 and 12 months. These findings support the use of transperitoneal laparoscopic radical prostatectomy, as our study patients exhibited significant benefits from this procedure.

## 1. Introduction

Laparoscopic radical prostatectomy (LRP) is the mainstay treatment of localized prostate cancer in some countries. The transperitoneal laparoscopic radical prostatectomy procedure was first reported in 1998 [1] and followed by the report of the extraperitoneal approach [2]. The two most common approaches are the transperitoneal and extraperitoneal routes, generally depending on surgeon preference. The advantage of the extraperitoneal approach is that the operation can be manipulated without intraperitoneal organ involvement, while the transperitoneal approach offers a larger working space and better visualization [3–6]. The previous studies showed comparable outcomes between both techniques, however, some surgeons switched between the approaches [7–9]. In this study, we aimed to compare the perioperative, functional, and oncological outcomes between the 2 approaches of laparoscopic radical prostatectomy.

## 2. Materials and Methods

### 2.1. Study Population

The study population was 266 patients with localized or locally advanced prostate cancer who underwent laparoscopic radical prostatectomy at Songklanagarind Hospital between April, 2008, and December, 2020, using transperitoneal (*n* = 98) or extraperitoneal (*n* = 168) surgery. The inclusion criteria for both operations were the same: diagnosed localized or locally advanced prostate cancer, no clinical lymph node or other metastasis, and body mass index ≤35 kg/m^2^. The exclusion criteria for both operations were comorbidities such as chronic obstructive pulmonary disease (COPD) due to concerns with the pneumoperitoneum and patients who did not complete a 2-yearfollow-up. All patients were followed up for clinical conditions, PSA, and functional outcomes. The surgeons in both groups had more experience in laparoscopic surgery in more than 50 cases. The study was reviewed and approved by the Institutional Review Board (REC no. 62-163-10-1).

### 2.2. Pathological Evaluation

All fine-needle biopsies and specimens were evaluated by a pathologist. Tumors at the inked margin were defined as positive surgical margins (PSM). The tumors were graded according to the Gleason score, and pathological staging was based on the TNM 2000 classification.

### 2.3. Outcomes

The following outcomes were used to compare the T-LRP with E-LRP. The perioperative parameters included were operative time (minutes), operative blood loss (milliliters), the rates of intraoperative and postoperative complications, and the time of postoperative catheterization (days). All outcomes were assessed at the patient level. The patients' continence and erection status were followed. “Continence” was defined as not using a pad, while incontinence was defined as requiring at least one pad per day. We evaluated the surgical margin status in all patients. Clinical conditions were assessed based on preoperative prostate-specific antigen levels and biopsy Gleason scores. Functional outcomes were assessed using the Expanded Prostate Cancer Index Composite (EPIC) questionnaire with 2 focus domains, urinary and sexual symptoms, with domain scores ranging from 0 to 100, with higher scores corresponding to better outcomes.

The patients completed the EPIC questionnaire preoperatively and at 3, 12, and 24 months postoperatively. The oncological outcomes followed were positive surgical margin status and biochemical recurrence. Positive margins were a binary variable. An inked surgical margin transected cancer, functional (erection and continence), and complication rates. A BCR was defined by two consecutive PSA levels of more than 0.2 ng/mL.

### 2.4. T-LRP Technique

The patient was placed supine in a 20° to 30° inclined Trendelenburg position after general anesthesia was administered. A sterile 20-French Foley catheter was inserted. An umbilical incision of 2 cm was made. Then successive dissections of the peritoneum were performed. Next, a 12-mm trocar was inserted into the peritoneal cavity. After a pneumoperitoneum was established at 12 to 15 mmHg, a camera was placed through this port. The other three ports and one assistant port were placed under vision. First, median umbilical ligaments on both sides were incised in a reversed “*U*” manner. Then, the endopelvic fascia was exposed and incised. Next, the dorsal vein complex was sealed with vessel sealing and bladder neck dissection was performed. After the catheter was removed, the seminal vesicles were dissected, and the vas deferens were cut off. On the superficial Denonvilliers fascia, the dissection of the prostate apex was dissected from the superficial Denonvilliers fascia, and the prostate could dissect after the junction of the prostate and proximal urethra was incised. The urethra-vesical anastomosis was performed without bladder neck preservation, sparing or reconstruction of the puboprostatic ligament, posterior reconstruction of the rhabdomyosphincter, and anterior retropubic suspension. Finally, drainage was placed in the pelvis, which was usually removed after the surgical wounds were closed. Pelvic lymph node dissection (PLD) was performed in all cases.

### 2.5. E-LRP Technique

The patient was placed supine in a 15° to 20° inclined Trendelenburg position. After the catheter was inserted, a 3 cm infraumbilical incision was made. Then successive dissections were performed until the posterior rectus sheath was exposed. The surgeon's middle finger was introduced behind the rectus muscles, and an extraperitoneal space was established by blunt digital dissection and balloon dilation. A camera was placed through the infraumbilical incision with a 12-mm trocar. A pneumoperitoneum was then established to be used for the transperitoneal approach. Next, two 5-mm ports were created and placed under vision. After the left iliac fossa was identified and dissected, another laparoscopic port was placed at the suprapubic area. Thus, 4 trocars (one for the camera, two surgical ports, and one for the assistant) were established. The prostatectomy was then performed basically in the same manner as with the TP approach. The urethra-vesical anastomosis was performed without bladder neck preservation, sparing or reconstruction of the puboprostatic ligament, posterior reconstruction of the rhabdomyosphincter, and anterior retropubic suspension. Pelvic lymph node dissection was performed in all cases.

### 2.6. Statistical Analysis

The results are reported as means and SDs or as numbers and percentages. The independent *t*-test was used to compare numerical values, while the Chi-square and Fisher's exact tests were employed to compare categorical outcome variables. The nonparametric Wilcoxon rank-sum test was used to determine differences in functional outcomes in the urinary and sexual domain scores and other continuous factors between the surgical approaches, while chi-square testing was utilized for categorical variables. Univariable and multivariable Cox regression analyses were used to identify factors predictive of BCR. All statistical analyses were conducted using the R programming version 4.2.1. Statistical significance was set at a *p* value of 0.05.

## 3. Results

266 men diagnosed with adenocarcinoma of the prostate were enrolled between April, 2008, and December, 2020, with 168 undergoing extraperitoneal laparoscopic radical prostatectomy (E-LRP) and 98 undergoing transperitoneal laparoscopic radical prostatectomy (T-LRP). Both groups of patients had comparable clinical characteristics, including age, body mass index(BMI), preoperative prostate-specific antigen levels, clinical T stage, Charlson comorbidity index, and pathologic outcomes (Table 1).

In terms of perioperative characteristics, the transperitoneal approach had lower overall operative time (22.5 min vs. 290 min, *p* < 0.001), lower blood loss (400 ml vs. 800 ml, *p* < 0.001), and shorter hospital stay (4 days vs. 7 days, *p* < 0.001). Urinary continence was comparable between the across extraperitoneal and transperitoneal methods at 3 and 24 months following surgery (3% vs. 1%; *p*=0.419, 85.1% vs. 83.7, respectively, *p*=0.899) but lower at 12 months in the extraperitoneal group (49.4% vs. 34.6%, respectively, *p*=0.028). At 3 months, the transperitoneal group had a better sexual performance with penile penetrance (36.7% vs. 15.5%, *p*=0.001) as shown in Table 2.

Perioperative complications were evaluated at early (<30 days POD) and late (>30 days POD) periods following the surgeries. Early complications (<30 days POD) were not different between the groups (11.3% vs. 14.3% in the E-LRP and T-LRP groups, respectively; *p*=0.09), while late complications (>30 d POD) were significantly lower in the T-LRP group (2% vs. 8.9% in the T-LRP and E-LRP groups, respectively; *p*=0.04), as shown in Table 3. We found 18 (10.7%) patients with early minor complications, including perineal pain, abdominal wall hematoma, and urinary leakage, in the E-LRP group, and 10 (10.2%) minor complications in the T-LRP group. One early major complication (0.6%) occurred in the E-LRP group and should stick with them, and there were four cases of urosepsis in the T-LRP group (4.1%). The postoperative complications using the modified Clavien Classification System were stratified into five grades. Between the two surgical approaches, we found that the E-LRP group had significantly higher rates of Clavien grade II and IIIb complications (5.4% vs. 3.4%; *p*=0.029, 9.5% vs. 7.1%; *p*=0.021, respectively), but the differences between the groups were not significantly different in the other complication grading. The most adjunctive procedure was hernioplasty had no difference in both groups (Table 3).

When examining postoperative pathologic data, we found that positive surgical margin rates were lower in the transperitoneal approach group (21.4% vs. 38.7% in the T-LRP and E-LRP groups, respectively; *p*=0.006). The biochemical recurrence occurred at 36.3% in the extraperitoneal approach group and 27.6% in the transperitoneal approach group (*p*=0.184). Other postoperative pathologic data are similar in both groups as shown in Table 4.

The EPIC questionnaire was used to assess functional outcomes at 3, 12, and 24 months after the surgeries. The urinary function scores were significantly higher in the T-LRP group at 3 and 12 months (55% vs. 50% 30; *p* < 0.001, 37.9% vs. 33.0%; *p* < 0.001, respectively) but at 24 months they were the same (72% vs. 72%; *p*=0.734), and the sexual function scores were also higher in the T-LRP group at 12 and 24 months (36% vs. 30%; *p* < 0.001, 41% vs. 34%; *p* < 0.001), while again being similar at 3 months post-surgery (Table 5).

### 3.1. Survival Outcomes

On univariable analysis, clinical T staging, preoperative prostatic specific antigen (PSA) level, biopsy Gleason score, Gleason grade group, National Comprehensive Cancer Network (NCCN) risk group, surgical margin, lymphovascular invasion(LVI), pathologic T stage, and nodal status were all significantly associated with biochemical recurrence (BCR) (*p* < 0.05), while age, body mass index (BMI), and surgical technique were not significantly associated (*p* > 0.05) and were excluded from further analysis. In the postoperative analysis, pGS had the highest significance in comparison with the other parameters in predicting BCR, with the highest hazard ratio (HR) of 8.33.

Multivariable Cox regression analysis showed the remaining variables, clinical T staging, marginal status, pathologic Gleason score, pathologic T stage, and pathological nodal status to be statistically significant and independently predictive of BCR (*p* < 0.05, Table 6).

**Figure 1:**
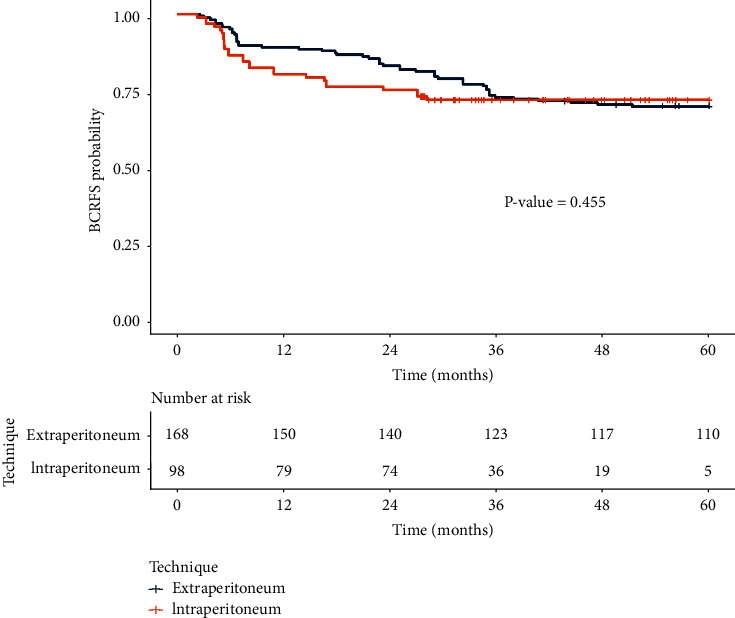
Kaplan–Meier survival curve of BCR-free survival postlaparoscopic radical prostatectomy between intraperitoneal and extraperitoneal approaches.

Figure 1 shows the Kaplan–Meier survival analysis of BCR-free survival between the transperitoneal and extraperitoneal approaches without a statistically significant difference (*p*=0.455)

## 4. Discussion

Laparoscopic radical prostatectomy (LRP) is now widely used to treat localized PCa in most developing nations. LRP provides the advantages of minimal trauma, minimal postoperative pain, and speedy recovery following surgery for localized or locally advanced prostate cancer. Recently, robot-assisted laparoscopic radical prostatectomy (RALRP) has shown unique advantages such as flexible operation equipment, three-dimensional vision, and a quick learning curve, which many facilities in industrialized countries have adopted [10]. PSM, urine continence, and sexual function have all shown better outcomes with RALRP than LRP, according to several systematic reviews and meta-analyses [11–14]. However, several studies have reported that RARP is more expensive than LRP due to the higher cost of surgical instruments [13, 15, 16]. There is still a considerable ongoing debate about the merits of T-LRP versus E-LRP. Issues regarding surgical effects and patient-related outcomes remain. While T-LRP is more popular, E-LRP has the advantages of no bowel contact and a faster return to normal oral intake. Both strategies have benefits and drawbacks. The absence of bowel contact in E-LRP reduces the risk of intra-abdominal organ harm. The downsides are a smaller operational field and a smaller view angle. The transperitoneal method is advantageous for extensive lymph node dissection and imaging.

In our study, the patient characteristics were similar between the E-LRP and T-LRP groups except for higher PSA in the T-LRP group, which may have been related to the biochemical recurrences differences postoperatively. The T-LRP surgeries had lower overall operative time (222.5 min vs. 290 min, *p* 0.001), similar to earlier studies which reported that T-LRP had bigger operative spaces and shorter operative times than E-LRP [17, 18].

In terms of EBL, the transperitoneal patients had less blood loss than the extraperitoneal patients (800 mL vs. 400 mL, respectively, *p* < 0.001). Some other studies comparing EBL between E-LRP and T-LRP reported discrepancies; however, they found less blood loss, for which a possible explanation is the ergonomic space [19–21]. One possibility is an ergonomic space of the intraperitoneal cavity can exert enough pressure on the surrounding tissue to suppress bleeding, and one possible explanation for the significant difference in blood loss is that in the transperitoneal group, a considerable amount of blood was retained among the bowel loops.

The postoperative length of stay was significantly longer in the E-LRP group (7 days vs. 4 days, *p* < 0.001), which might be explained by the disadvantages of E-LRP performed in the early period of minimally invasive surgery. Early sexual intercourse with penetration at 3 months was higher in the T-LRP (36.7% vs. 15.5%, *p* 0.001), although the intraperitoneal group had a considerably better ability to penetrate. This indicates that differences in results between different surgical approaches may be related to nerve-sparing considerations in the surgical technique, and long-term evaluations are needed.

The recovery of urinary control is an important factor to consider when assessing the functional prognosis following RP. Urinary continence (no pads) was not different between the T-LRP and E-LRP groups at 3 and 24 months after surgery but was superior in the T-LRP group at 12 months(1% vs. 3%; *p*=0.419, 85.1 vs. 83.7%; *p*=0.889, 47.4% vs. 34.6%, respectively; *p*=0.028). We believe that there are more adverse postoperative influences on the celiac organs due to various factors related to the entering of the peritoneal cavity, such as the intraperitoneal insufflation of CO_2_, [6] which might in turn lead to higher rates of overall postoperative complications in the T-LRP group, indicating a stable recovery of urinary continence without increasing the incidence of postoperative urinary incontinence. Asimakopoulos et al. [22] reported urinary control rates of 63.3%, 75.0%, and 83.3% at 3 months, 6 months, and 1 year after LRP, respectively. Ploussard et al. [23] found that at 3 months, 6 months, and 1 year, the urine control rates of 1377 patients with LRP were 39.4%, 58.9%, and 68.5%, respectively. Porpiglia et al. [24] reported continence rates at 3 months, 6 months, and 1 year were 61.6%, 73.3%, and 83.3%, respectively. In our study, early urine continence rates were similar for both the extraperitoneal and transperitoneal methods at 3 and 12 months (21.5% and 26.4%, *p*=0.34). The EPIC questionnaire was used to assess functional outcomes at 3,12, and 24 months after surgery, and found that urinary function was significantly better in the T-LRP group at 3 and 12 months (*p* < 0.001), but at 24 months, there was no difference (*p*=0.734), and sexual function was also better in the T-LRP group at 12 and 24 months (*p*=0.001). A higher rate of lymphoceles was found with the extraperitoneal approach than with the transperitoneal approach.

PSM is a predictor of tumor progression that can be avoided by careful patient selection and surgical technique [21], which is closely related to PSA biochemical recurrence and postoperative adjuvant treatment [25]. For perioperative oncological outcomes, our results suggest that the rate of positive surgical margins was higher in the E-LRP group (38.7% vs. 21.4%; *p*=0.006). Postoperative pathological outcomes were closely linked to PSM and GS. This was different from the previous study of Hakimi et al. [26], which compared PSM in LRP vs. RALRP and E-LRP vs. T-LRP, and found no statistically significant differences. BCR is another critical index of oncological outcomes closely related to PSM. Our study found that the BCR rates were not significantly different between the groups (36.3% in E-LRP and 27.6% in E-LRP; *p*=0.184) but higher compared with recent studies. However, the relatively high PSM and biochemical recurrence rates in this series should not be ignored. We also reviewed the biopsy GSs and preoperative PSAs of all the study patients and found that most patients were at or above intermediate risk. Furthermore, the extraprostatic extension rate suggested similar results in postoperative pathology. No significant differences were observed in postoperative GSs between the two groups. Moreover, the experience of the surgeon may affect the outcomes of prostatectomy; the previous study reported assessing the quality of RARP training on a multicentric dataset. Several findings are noteworthy. The proficiency score provides a quick and detailed metric for summarizing major perioperative and pathologic outcomes of interest for RARP performed by naïve surgeons [27, 28]. However, our study has similar experience surgeons in both groups, which had a less biased impact on surgical outcomes.

The study had some limitations which should be noted. The lack of randomization may have introduced a selection bias based on patient or surgeon preferences. It is crucial to need further studies to look at longer-term outcomes such as biochemical recurrence and comorbidities, as well as variations in the two surgical procedures. The extraperitoneal procedure exhibited a similar positive surgical margin rate to the intraperitoneal method. The EPIC questionnaire in our study showed adequate erectile function following extraperitoneal treatment in a few instances. Moreover, differences in outcomes between different surgical techniques may not be apparent until long-termfollow-up. For further research, a large prospective randomized controlled study with long-termfollow-up is required.

## 5. Conclusion

Transperitoneal laparoscopic radical prostatectomy was found to be superior to extraperitoneal radical prostatectomy in terms of perioperative outcomes such as decreased operative time, decreased blood loss, shorter hospital stays, lower rate of positive surgical margin, and improved sexual function postoperatively. However, urinary and sexual function evaluations across all time points in both groups did not show any differences.

## Figures and Tables

**Table 1:** Demographics and clinical characteristics of the study population between extraperitoneal and transperitoneal approach.

Characteristics	Group 1: E-LRP (*n* = 168)	Group 2: T-LRP (*n* = 98)	*p* value
Age (years, mean ± SD)	68.4 (6.1)	69.1 (6)	0.346
BMI (median, IQR)	24 (22.9, 26.5)	24.8 (22.6, 26.3)	0.811
Charlson Comorbidity Index	1 (0–6)	1 (0–7)	0.81
PSA (ng/mL)	12.5 (8.1, 22.9)	16.6 (10.7, 24.4)	0.005^*∗*^
*Clinical T stage-n (%)*			0.095
1b-2a	14 (8.3)	13 (13.3)	
2b	11 (6.5)	8 (8.2)	
2c	98 (58.3)	40 (40.8)	
3a	14 (8.3)	15 (15.3)	
3b	30 (17.9)	22 (22.4)	
4	1 (0.6)	0 (0)	
*Gleason score-n (%)*			0.228
6 (3 + 3)	62 (36.9)	23 (23.5)	
7 (3 + 4)	45 (26.8)	33 (33.7)	
7 (4 + 3)	37 (22)	24 (24.5)	
8 (4 + 4)	10 (6)	6 (6.1)	
9 (5 + 4)	14 (8.3)	12 (12.2)	
*Nerve sparing (NS)*			0.448
Non	145 (86.3)	79 (80.6)	
Unilateral	19 (11.3)	15 (15.3)	
Bilateral	4 (2.4)	4 (4.1)	

PSA: prostatic specific antigen and BMI: body mass index.

**Table 2:** Comparison of perioperative data.

	Group 1: E-LRP (*n* = 168)	Group 2: T-LRP (*n* = 98)	*p* value
Overall operative time, min (IQR)	290 (245, 385)	222.5 (175, 255)	<0.001
Blood loss (mL)	800 (500, 1200)	400 (300, 537.5)	<0.001
Time to discharge, day (IQR)	7 (7, 7)	4 (4, 5)	<0.001
Erectile function			
Sexual intercourse at 3 months			0.001
No	142 (84.5)	62 (63.3)	
Penetrate	26 (15.5)	36 (36.7)	
Continence at 3 months			0.419
No pad	5 (3)	1 (1)	
≥1 pad a day	163 (97)	97 (99)	
Continence at 12 months			0.028
No pad	83 (49.4)	34 (34.6)	
≥1 pad a day	85 (50.6)	64 (65.3)	
Continence at 24 months			0.889
No pad	143 (85.1)	82 (83.7)	
≥1 pad a day	25 (14.9)	16 (16.3)	

IQR; inter quartile range.

**Table 3:** Postoperative complications.

	Group 1: E-LRP (*n* = 168)	Group 2: T-LRP (*n* = 98)	*p* value
Early complication (before 30^th^ POD)	19 (11.3)	14 (14.3)	0.09
*Minor-n (%)* Perineal pain Abdominal wall hematoma Urinary leakage	18 (10.7)	10 (10.2)	0.13
*Major-n (%)* Urinary sepsis	1 (0.6)	4 (4.1)	0.018
Late complication (after 30 d)	15 (8.9)	2 (2)	0.04
*Minor-n%* Bladder neck stricture Urethral meatus stricture UTI	12 (7.1)	2 (2)	0.006
*Major-n (%)* Reoperation Death	3 (1.8)	0	0.299
*Postoperative complications*	30 (17.9)	25 (25.5)	
Grade I	3 (1.8)	7 (2.6)	0.429
Grade II	9 (5.4)	9 (3.4)	0.029
Grade IIIa	2 (1.2)	2 (0.8)	0.533
Grade IIIb	16 (9.5)	7 (7.1)	0.021
Grade IVa	0	0	
*Adjunctive procedure*			0.768
No	161 (95.8)	97 (99)	
Hernioplasty	5 (3)	1 (1)	
Appendectomy	1 (0.6)	0 (0)	
Closure colostomy	1 (0.6)	0 (0)	

**Table 4:** Postoperative pathologic data.

Variable	Group 1: E-LRP (*n* = 168)	Group 2: T-LRP (*n* = 98)	*p* value
*T stage*			0.377
2a	17 (10.1)	14 (14.3)	
2b	11 (6.5)	10 (10.2)	
2c	90 (53.6)	40 (40.8)	
3a	17 (10.1)	13 (13.3)	
3b	32 (19)	21 (21.4)	
4	1 (0.6)	0 (0)	
*N stage*			0.846
0	160 (95.2)	92 (93.9)	
1	8 (4.8)	6 (6.1)	
Positive surgical margin	65 (38.7)	21 (21.4)	0.006
LVI	25 (14.9)	21 (21.4)	0.232
Biochemical recurrence	61 (36.3)	27 (27.6)	0.184

LVI; lymphovascular invasion.

**Table 5:** Urinary and sexual function evaluation across time points by surgery type at preoperative 3 months, 12 months, and 24 months.

	Range	*Baseline*			*3 months*			*12 months*			*24 months*		
		Group 1: E-LRP (*n* = 168)	Group 2: T-LRP (*n* = 98)	*p*value	Group 1: E-LRP (*n* = 168)	Group 2: T-LRP (*n* = 98)	*p*value	Group 1: E-LRP (*n* = 168)	Group 2: T-LRP (*n* = 98)	*p*value	Group 1: E-LRP (*n* = 168)	Group 2: T-LRP (*n* = 98)	*p*value
Urinary function													
EPIC—urinary domain	0–100	80 (78.8, 85)	80 (80, 85)	0.611	50 (50, 60)	55 (50, 60)	< 0.001	60 (55, 65.2)	65 (60, 70)	<0.001	72 (70, 78)	72 (70, 79.5)	0.734
Sexual function													
EPIC—sexual domain	0–100	55.5 (48, 60)	60 (50, 70)	0.005	25 (22, 30)	30 (20, 40)	0.078	30 (25, 36.2)	36 (26.5, 50)	<0.001	34 (28, 42)	41 (32, 60)	<0.001

EPIC = expanded prostate cancer index composite.

**Table 6:** Cox univariable and multivariable analyses.

Variable	*Univariable analysis*			*Multivariable analysis*		
	HR	95% Cl	*p* value	HR	95% Cl	*p* value
*Age (years)*						
<70	Ref		0.083			0.018
≥70	1.45	0.95, 2.2		1.71	1.1, 2.68	
*Clinical T stage*						
T1-T2b	Ref		<0.001			<0.001
T2c	0.77	0.34, 1.74		0.53	0.22, 1.27	
T3a	1.6	0.62, 4.12		0.95	0.33, 2.72	
T3b-T4	4.01	1.78, 9.05		1.82	0.65, 5.06	
*Initial PSA (ng/mL)*						
≤10	Ref		<0.001	Ref		0.609
10–20	1.4	0.79, 2.48		1.24	0.69, 2.26	
>20	2.62	1.55, 4.46		1.36	0.73, 2.55	
*Gleason grade group*						
1	Ref		<0.001			
2	1.65	0.84, 3.25				
3	2.91	1.53, 5.53				
4	4.58	2, 10.48				
5	8.33	4.25, 16.33				
*BMI (mg/m * ^*2*^ * )*						
<25	Ref		0.313			
≥25	1.24	0.82, 1.89				
*NCCN risk classification*						
Low	Ref		<0.001			
Intermediate	0.81	0.24, 2.7				
High	1.62	0.49, 5.37				
Very high	5.64	1.73, 18.32				
*Margin status*						
Negative	Ref		<0.001	Ref		0.046
Positive	2.76	1.78–3.85		1.52	1.01–2.29	
*LVI status*						
Negative	Ref		<0.001			
Positive	2.33	1.45, 3.73				
*Pathologic Gleason score*						
6	Ref		<0.001			0.021
7	1.64	0.92, 2.91		1.39	0.74, 2.61	
≥8	5.17	2.81, 9.5		2.71	1.28, 5.74	
*pT stage*						
1-2c	Ref		<0.001	Ref		<0.001
3a	1.93	0.99, 3.78		2.78	1.6–4.83	
3b-4	5.83	3.7, 9.18		5.15	3.07–8.64	
* pN stage*						
0	Ref		0.006	Ref		0.028
1	3.06	1.53, 6.12		3.1	1.65–5.81	
*Technique*						
Intraperitoneal	Ref					
Transperitoneal	1.12	0.7, 1.79	0.638			

BMI; body mass index, NCCN; National Comprehensive Cancer Network, and LVI; lymphovascular invasion.

## Data Availability

The data that support the findings of this study are available from the corresponding author upon reasonable request.
